# Identification of discriminatory antibiotic resistance genes among environmental resistomes using extremely randomized tree algorithm

**DOI:** 10.1186/s40168-019-0735-1

**Published:** 2019-08-29

**Authors:** Suraj Gupta, Gustavo Arango-Argoty, Liqing Zhang, Amy Pruden, Peter Vikesland

**Affiliations:** 1 The Interdisciplinary PhD Program in Genetics, Bioinformatics, and Computational Biology, Virginia Tech, Blacksburg, VA 24061 USA; 2 Department of Computer Science, Virginia Tech, Blacksburg, VA 24061 USA; 3 Via Department of Civil and Environmental Engineering, Virginia Tech, Blacksburg, VA 24061 USA

**Keywords:** Antibiotic resistance genes, Aquatic environments, Ensemble learning, Extremely randomized trees, Wastewater, Surveillance

## Abstract

**Background:**

The interconnectivities of built and natural environments can serve as conduits for the proliferation and dissemination of antibiotic resistance genes (ARGs). Several studies have compared the broad spectrum of ARGs (i.e., “resistomes”) in various environmental compartments, but there is a need to identify unique ARG occurrence patterns (i.e., “discriminatory ARGs”), characteristic of each environment. Such an approach will help to identify factors influencing ARG proliferation, facilitate development of relative comparisons of the ARGs distinguishing various environments, and help pave the way towards ranking environments based on their likelihood of contributing to the spread of clinically relevant antibiotic resistance. Here we formulate and demonstrate an approach using an extremely randomized tree (ERT) algorithm combined with a Bayesian optimization technique to capture ARG variability in environmental samples and identify the discriminatory ARGs. The potential of ERT for identifying discriminatory ARGs was first evaluated using in silico metagenomic datasets (simulated metagenomic Illumina sequencing data) with known variability. The application of ERT was then demonstrated through analyses using publicly available and in-house metagenomic datasets associated with (1) different aquatic habitats (e.g., river, wastewater influent, hospital effluent, and dairy farm effluent) to compare resistomes between distinct environments and (2) different river samples (i.e., Amazon, Kalamas, and Cam Rivers) to compare resistome characteristics of similar environments.

**Results:**

The approach was found to readily identify discriminatory ARGs in the in silico datasets. Also, it was not found to be biased towards ARGs with high relative abundance, which is a common limitation of feature projection methods, and instead only captured those ARGs that elicited significant profiles. Analyses of publicly available metagenomic datasets further demonstrated that the ERT approach can effectively differentiate real-world environmental samples and identify discriminatory ARGs based on pre-defined categorizing schemes.

**Conclusions:**

Here a new methodology was formulated to characterize and compare variances in ARG profiles between metagenomic data sets derived from similar/dissimilar environments. Specifically, identification of discriminatory ARGs among samples representing various environments can be identified based on factors of interest. The methodology could prove to be a particularly useful tool for ARG surveillance and the assessment of the effectiveness of strategies for mitigating the spread of antibiotic resistance. The python package is hosted in the Git repository: https://github.com/gaarangoa/ExtrARG

**Electronic supplementary material:**

The online version of this article (10.1186/s40168-019-0735-1) contains supplementary material, which is available to authorized users.

## Background

As recognized by the World Health Organization (WHO) and other national and international bodies, antibiotic resistance poses a serious threat to public health and is a major impediment to the application of antibiotics for effective infectious disease treatment [1, 2]. Substantial effort has been directed towards understanding the factors that contribute to the spread of resistance and the means to control it. While antibiotic resistance has likely existed since bacteria and their competitors first evolved, the development, mass production, and widespread use of antibiotics in humans and livestock is understood to have sped up evolution of antibiotic resistance, leading to new types, higher abundances, and enhanced horizontal transfer of antibiotic resistance genes (ARGs) among microbial populations. Thus, it is critical to identify how human activities and interventions influence the mechanisms by which resistance evolves and spreads and alters occurrence relative to the “natural” background condition [3]. Notably, selective pressures exerted by antibiotic residues and other co-selecting factors, such as metals and surfactants, can act to sustain and exacerbate the selection and spread of ARGs [4, 5].

Of greatest concern is the carriage of ARGs by clinical pathogens, which severely endangers the effective use of antibiotics as human and veterinary medicines [6, 7]. Pathogenic bacteria have been documented to be capable of obtaining ARGs from non-pathogenic bacteria [8]. In particular, soil and other natural environments are known to contain a rich diversity of microorganisms and have been described as a reservoir and source of ARGs [9, 10]. Under favorable conditions, these ARGs can be transferred to pathogenic bacteria via horizontal gene transfer, thus extending resistance to new bacteria [11]. Such processes are extremely difficult, if not impossible, to monitor in real time, and thus, there is a need to develop tools to systematically and objectively assess how anthropogenic impacts, such as inputs of resistant bacteria, ARGs, and selective agents, collectively shape the “resistomes” (i.e., the full complement of ARGs in a system [12]) of affected environments.

Several studies have identified aquatic environments as key conduits of ARGs, where anthropogenic inputs interact with resident microbes, with a feedback loop returning back to human exposure via affected drinking water, recreational water, food, and aerosols [13, 14]. High ARG loadings in wastewater discharges have been found to exert a strong influence on aquatic environments, such as rivers and surface waters, and can aid in augmenting the ARG pool [15, 16]. In particular, extensive use of antibiotics in clinical and agricultural settings has established hospital wastewater and farm wastewater effluents as potential “hot spots” for the evolution and spread of antibiotic resistance [17–19]. ARG profiles and patterns in surface waters and river water, which are often treated to use for drinking purposes, are profoundly influenced by agricultural and wastewater inputs [20–24]. Assessing the human health risk represented by the ARG content of aquatic environments remains a crucial endeavor. Wastewater treatment plants (WWTPs) serve as a critical node for either mitigation or dissemination of ARGs. Wastewater from various sources may contain antibiotics and other bacterial stressors at varying concentrations depending on the local antibiotic consumption/usage pattern, which could lead to different microbial communities, ARG profiles, and ARG loadings [25]. Wastewater influents and sludge discharges are often found to be rich in ARGs and other co-selecting agents [26, 27]. Hence, there has been increased attention placed on the characterization of WWTP influents (i.e., sewage) and effluents in the context of antibiotic resistance.

Various methods have been applied towards environmental bacterial resistance surveillance, but there remains a lack of consensus on a standardized approach. Molecular methods are often favored due to a lack of representation of the full resistome by culture-based approaches. In particular, quantitative polymerase chain reaction (qPCR) has been widely used to profile and quantify a wide array of ARGs in environmental samples [28, 29]. However, qPCR requires a priori selection of targets and thus may overlook the key ARGs within a given environment [30]. Over the past decade, shotgun metagenomic sequencing has emerged as a powerful tool that can reveal the broad spectrum of ARGs present in clinical and environmental samples [31, 32]. Recent studies have used metagenomics to characterize and compare ARG profiles in different urban water systems and natural water bodies [33] and between different influent and effluent WWTP samples [34, 35]. However, analysis of metagenomic data is challenging, and to date, there are no standardized means for assessing and comparing resistome characteristic of a given sample or environment. Most commonly, metagenomic analysis has primarily employed feature projection methods, such as principal component analysis (PCA), principal coordinate analysis (PCoA), and non-metric multi-dimensional scaling (NMDS) [36]. A major limitation of these analyses is that they only provide measures of similarity or dissimilarity between samples, rather than identifying the actual ARGs that drive the observed differences. Due to the costs involved, metagenomic data sets are also often limited [37], which can further complicate analysis and decrease confidence in observed differences. In particular, the highly correlated nature of the variables in genomic data renders the independent assumptions required by many statistical models invalid. Statistical test-based tools such as LefSe [38] or DESeq [39] are readily used for identifying differentially abundant features, but come with their own limitations. Some of these methods often assume an underlying distribution of the data that may not be accurate for metagenomic data [40]. Machine learning techniques are emerging as a rapid and powerful way to capture such specific patterns and observations. As currently applied, discriminatory features are identified as those found to be relevant in building the corresponding machine learning model. However, this approach is empirical and the requirement of a user-provided threshold has a potential to introduce bias [41]. The prevalent automatized methods to select discriminant features work by recursively selecting the feature set and estimating model accuracy also known as wrapper methods. Unfortunately, such methods can be impractically slow when dealing with large and sparse datasets [42], such as those characteristics of metagenomic data. Thus, there is a need for analytical approaches that can appropriately account for such limitations and biases, that facilitate identification of key ARGs characteristic of a given sample or environment, and that identify the corresponding dissimilarities relative to other samples.

Ensemble learning methods have recently been introduced as a means of managing complex multi-dimensional data sets, such as those derived from metagenomic sequencing. In particular, the extremely randomized tree (ERT) algorithm, enabled by the emerging field of machine learning, is growing in popularity [43]. ERT uses a similar approach to random forests (RF) [44] to build an ensemble of trees, but with two major differences: (1) instead of using bagging features, it employs full datasets to grow and learn the trees, and (2) the node split is picked randomly, as compared to RF, where best splits are chosen within the random subset and are sampled. The ERT algorithm is especially efficient in handling correlations and interactions among variables and provides effective data inference. ERT algorithms can also serve to rank features by variable importance measures and can improve differentiation of classes based on the feature variables. This property of ERT algorithms holds particular promise for identifying discriminatory ARGs that could be used to characterize the differences among samples according to their groups. However, like other machine learning methods, ERT requires the optimization of parameters to improve its performance. The selection of such parameter values is not straightforward and is dependent on the data that is being processed. A Bayesian-based optimization strategy [45, 46] could potentially overcome this challenge by providing a means to tune the parameters of the ERT to maximize discriminatory ARG identification.

The objective of this study was to formulate an ERT methodology for identifying discriminatory ARGs among different environmental compartments based on their corresponding shotgun metagenomic sequencing data. The potential of ERT for identifying discriminatory ARGs was first evaluated using in silico metagenomic datasets (simulated metagenomic Illumina sequencing data) with known variability. The application of ERT was then demonstrated through analyses using publicly available metagenomic datasets associated with (1) different aquatic habitats (e.g., river, wastewater influent, hospital effluent, and dairy farm effluent) to compare resistomes among distinct environments and (2) different river samples (i.e., Amazon, Kalamas, and Cam Rivers) to compare resistome characteristics of similar environments. Cluster analysis was done by estimating silhouette coefficients and Bray-Curtis similarities to quantitatively validate the performance of the ERT algorithm. The overall ERT approach holds promise for improving ARG surveillance in the environment and can aid in identifying sources and mechanisms of the spread of antibiotic resistance and assessing strategies for mitigation.

## Implementation

### The extremely randomized tree algorithm

The extremely randomized tree (ERT) algorithm is a tree-based ensemble method that is traditionally used for supervised classification and regression problems. The ensemble method is a process by which the outcomes from many decision trees are averaged to obtain a final output [47, 48]. ERT is used to deduce useful information from a labeled set of data. The labeled dataset contains “features” (also called attributes) and “classes” (or groups). Simply put, attributes are a set of parameters that together describe an object. For example, shape, taste, and color are all attributes that could describe a fruit. Accordingly, such attributes could be applied towards categorizing the fruits into different groups (e.g., apples and oranges). In the context of the present study, the attributes applied were relative abundances of the resistance genes (e.g., 16S rRNA gene normalized ARG abundances) and the groups are user-defined labels (e.g., sampling location, environments). The objective of the ERT algorithm was to map the resistance genes against the group labels and identify ARGs associated with different groups.

The ERT algorithm was constructed using an ensemble of Classification and Regression Trees (CART) [49]. These trees are grown by splitting the input dataset into subsets using simple decision rules deduced from the attribute information. The decision based on the ensemble reduces the variance of the model, without increasing bias, yielding more accurate classification. This technique largely overcomes overfitting problems associated with single classification tree methods. A key difference between ERT and other tree-based ensemble approaches is that it splits nodes using randomly generated cut-points for each feature. The randomness in choosing cut-point thresholds of the attributes reduces variance. The introduction of randomness in selecting the cut-point threshold and attributes reduces the variance effectively when combined with ensemble averaging. Another difference relative to other tree-based approaches is that ERT uses the full dataset to build the trees, whereas other methods adopt a bootstrapping approach to sample the dataset. In the bootstrapping method, only a portion of the data set is used to make the trees and this could lead to high bias in the resulting classification. Using the entire dataset helps to further reduce bias.

Ultimately, the ERT algorithm ranks the attributes based on their Gini importance to identify discriminatory ARGs. The Gini importance score indicates those attributes that are most efficient at effectively classifying the groups that contribute the most towards building strong decision trees [50, 51].

### Data preprocessing and labeling

After retrieving ARG abundances computed from MetaStorm [52], the samples were grouped and labeled. The labels were based on the desired classification scheme. Additional file 1: Figure S1 illustrates the labeling of different metagenomes based on user-defined group labels.

#### In silico datasets

Six artificial metagenomic datasets (of 1,000,000 reads) were generated using InsilicoSeq—a Python software package [53]. InSilicoSeq is a sequencing simulator that simulates metagenomic Illumina sequencing data from given genomes. We used 21 bacterial genomes and the default pre-computed error model to produce a typical metagenomic dataset for the Illumina Hiseq platform (for more information, see Additional file 2). These datasets were used to benchmark our methodology. The datasets were randomly divided into two groups (i.e., “A” and “B”), with each group containing three samples. To synthesize known differences among these groups, the reads of three randomly selected ARGs (*sul*1, *tet*(W), *erm*B) were added to the simulated datasets in varying and known proportions. *sul*1 was in high abundance in group “A” samples when compared with group B. *tet*(W) was in high abundance in group B samples in comparison with group A. *erm*B was added in high abundance, but with little variation among the groups.

#### Labeling for analysis 1

Metagenomic data sets extracted from public databases were selected to represent a cross section of a broad range of aquatic environments, including river, dairy farm, WWTP influent, and hospital effluents (Tables 1 and 2). To differentiate these samples, dairy farm effluents were labeled as “farm”, hospital effluents were labeled as “hospital,” and wastewater influent samples were labeled as “influents.” The Kalamas River and Cam River samples were labeled as “river,” while Amazon River plume samples were maintained in a separate group as “Amazon River plume” due to the expected minimal human intervention in the Amazonian region. The idea underlying this demarcation was to broadly analyze differences among the environments that are closely impacted by human activities from those environments that are expected to be relatively pristine.

**Table 1:** Metadata of different environmental samples obtained from public databases

Sample	Biomes	Sampling location/region	Description	Database	Accession number	Total number of reads	DNA extraction kit/method	Sequencing platform	Reference
ARP 1 ARP 2 ARP 3	Amazon River plume	Western tropical North Atlantic Ocean	Samples were collected at Amazon River plume	NCBI_SRA	SRR1185414 SRR1186214 SRR1199271	1,724,868 1,304,682 1,277,913	Method proposed by [54]	Illumina Genome Analyzer IIx	[55, 56]
KR 1 KR 2 KR 3	Kalamas River	Epirus region of Greece	Samples were collected from the Kalamas River	NCBI_SRA	SRR3098756 SRR3098759 SRR3098769	10,792,071 7,698,589 5,351,255	Mo Bio Power Soil Kit (Mo Bio Inc. Carlsbad, CA, USA)	Illumina HiSeq 2500	[57]
DF 1 DF 2 DF 3	Dairy farm effluent	Cambridge, UK	Samples were collected from the effluent lagoon of a dairy farm	EMBL-EBI	ERR1193297 ERR1193298 ERR1193301	23,154,788 82,819,396 26,421,627	Meta-G-Nome DNA Isolation Kit, Epicentre)	Illumina HiSeq 2000	[19]
HE 1 HE 2 HE 3	Hospital effluent	Cambridge, UK	Samples were collected from the combined wastewater effluents of the main wards of university hospital	EMBL-EBI	ERR1191817 ERR1191818 ERR1191819	30,295,299 22,689,323 50,303,545			
CR 1 CR 2 CR 3	Cam River	Cambridge, UK	Samples were collected within the River Cam catchment	EMBL-EBI	ERR1193292 ERR1193293 ERR1193294	17,899,008 68,902,092 43,078,304			
HE 4	Hospital effluent	Singapore	Sample was collected from general ward	NCBI-SRA	SRR5997540	2,257,389	PowerWater DNA Isolation Kit (Mo Bio Lab, Inc, CA)	Illumina HiSeq 2500	[58]
HE 5 HE 6	Hospital effluent	Singapore	Samples were collected from clinical isolation ward	NCBI-SRA	SRR5997541 SRR5997548	1,927,227 2,090,859

**Table 2:** Sampling information: WWTP influent samples

Sample ID	Sample type	Sampling country	Coordinates	Location	Sampling date	Total reads	Annotated read
IN 1	Influent	India	13.036238, 80.193738	Chennai	10 Mar. 2016	13,045,504	15,421
IN 2	Influent	USA	37.201889, − 76.447378	Christiansburg	19 Jan. 2017	13,460,770	11,776
IN 3	Influent	Philippines	14.592113, 121.058931	Mandalayong City	29 Nov. 2016	14,332,977	20,267
IN 4	Influent	Switzerland	47.405586, 8.597585	Zurich	18 May 2016	15,314,202	18,311
IN 5	Influent	Sweden	57.704713, 12.926666	Boras	8 Jun. 2016	11,801,763	10,149
IN 6	Influent	Hong Kong	22.406709, 114.213706	Hong Kong	14 Jul. 2016	15,763,560	14,979

#### Labeling for analysis 2

Deeper analysis of the river and similar environments was achieved by grouping samples by their respective rivers in order to identify the corresponding discriminatory ARGs and to compare resistome characteristic of riverine environments. The samples were labeled based on their respective rivers: “Amazon,” “Cam,” and “Kalamas.”

### Step-wise execution of ERT

#### The ExtraTreesClassifier

The ERT algorithm was applied to the labeled datasets using Python (3.2.5). The scikit-learn pre-built classifier, ExtraTreesClassifier, was used to build the ensemble and to calculate Gini importance scores. The number of estimators was set at a default value of 1000. The algorithm subsequently provides a list of attributes (i.e., ARGs) best suited for discriminating groups.

#### Identification of discriminatory ARGs using the ExtraTreesClassifier

The input dataset consisted of an abundance matrix where the “rows” represent the ARG abundances and the “columns” represent the samples. The system takes this matrix and the group labels as the input and returns the list of ARGs with their individual Gini importance (Additional file 1: Figure S2).

**Fig. 1:**
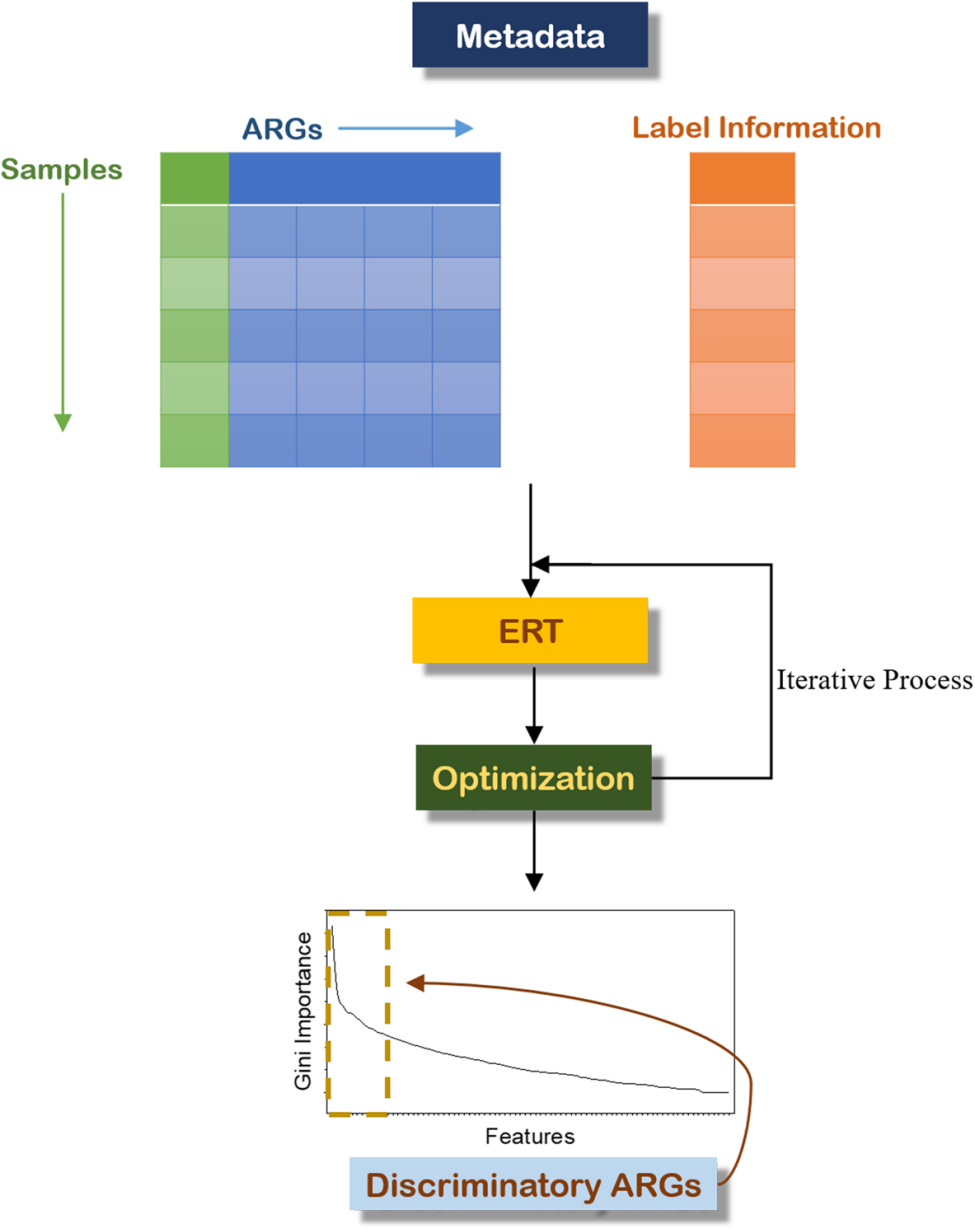
Computational pipeline for the selection of discriminatory ARGs

To improve the accuracy of the discriminatory ARG identification, the parameters of the ERT were tuned by using the Bayesian optimization approach [45, 59]. This method enables automatic identification of the parameters of the ERT relative to the input data. Specifically, the number of estimators and the importance cutoff used to determine the most relevant ARGs were optimized. By default, the algorithm runs through 50 iterations to identify the parameters that maximize segregation between the groups. The number of estimators was observed to range from 800 to 1000, and the Gini importance ranged from 10^−5^ to 10^−2^ based on the observation of the samples analyzed in this study. During each iteration, ARGs are potentially discarded due to their lack of importance. For instance, if the importance cutoff was set to 10^−3^, all ARGs below that value were discarded. The remaining ARGs were then fed into the ERT, and the predicted labels were compared to the actual labels using a customized loss function. This scoring function consists of the Rand index score, which computes the similarity among clusters adjusted to random chance [60]. Thus, values close to 0 are considered to be from random labels, whereas values close to 1 are considered to be identical to the true group labels [61, 62]. In the end, this step yields the optimum number of discriminatory ARGs for the specific analysis of interest. Figure 1 provides a schematic of the methodology.

#### Clustering

Group-average hierarchical clustering was obtained using PRIMER-E (v6). Cluster quality was evaluated by estimating silhouette coefficients and Bray-Curtis similarities to quantitatively validate the performance of the ERT algorithm [63]. The silhouette coefficient shows how well a sample is clustered to its correct cluster label compared to other clusters. The score ranges from − 1 to 1, where higher scores indicate better cluster assignment. Further, the dataset containing only the discriminatory ARGs was executed using a R code to generate a heatmap projecting the relative gene abundances. The library used for heatmap construction was *Complex Heatmap* [64].

## Results

### In silico metagenomic dataset

**Fig. 2:**
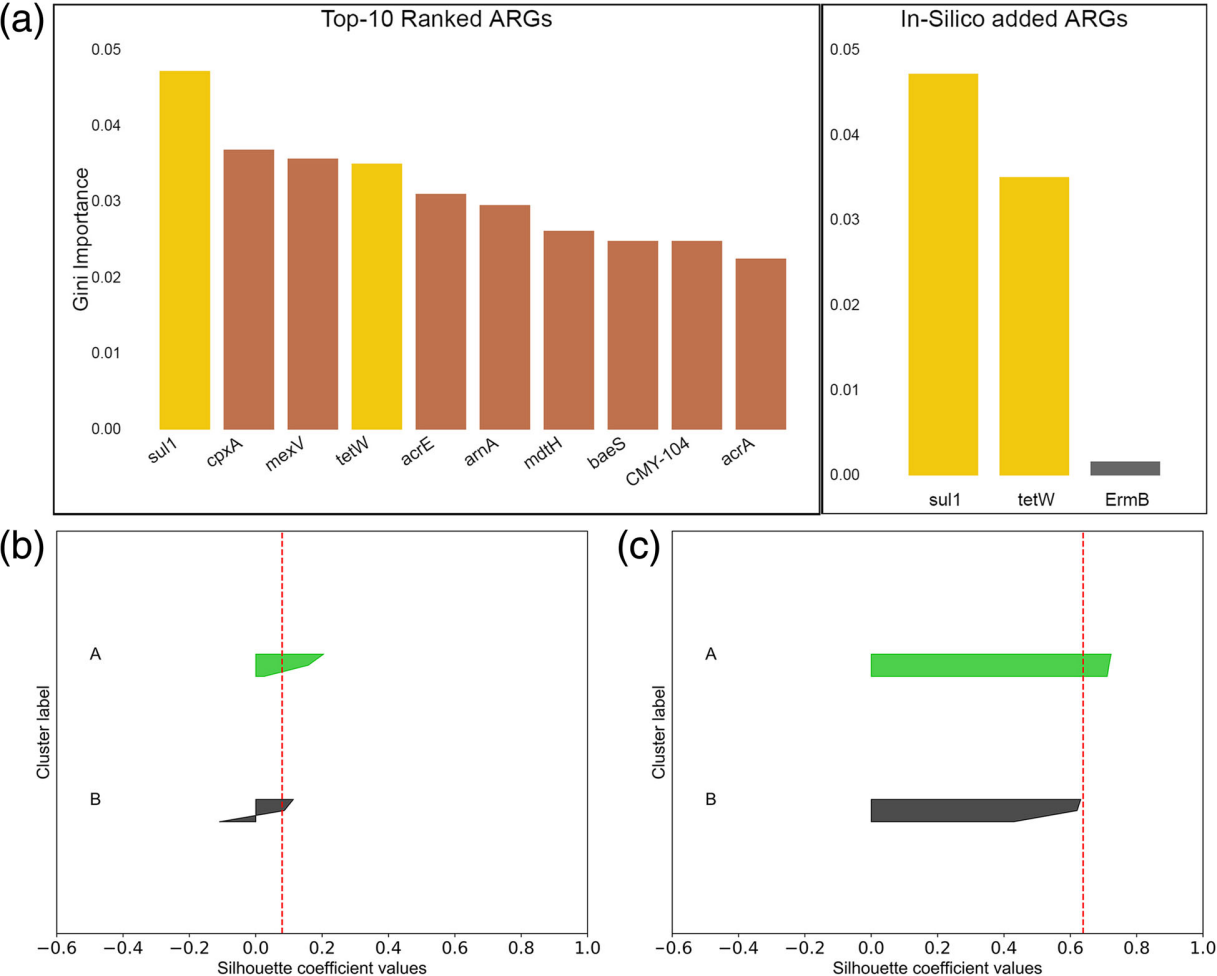
**a** (Left) Gini importance of the identified top 10 discriminatory ARGs. (Right) Gini importance of the ARGs (*sul*1, *tet*(W), *erm*B) added in the known variations to the in silico datasets (simulated metagenomic Illumina sequencing data generated using InSilicoSeq). **b** Silhouette plot for in silico samples using all the annotated ARGs. **c** Silhouette plot for in silico samples using the discriminatory ARGs

The potential and the limitations of ERT were first examined using in silico metagenomic datasets, within which three ARGs (*sul*1, *tet*(W), and *erm*B) with known abundances were embedded. Based on intentional manipulation of their relative abundances among the hypothetical groups generated in silico, ERT was expected to identify *sul*1 and *tet*(W) as discriminatory, but *erm*B as non-discriminatory. It was observed that ERT was correctly able to identify the discriminatory ARGs among the two labeled groups, i.e., “A” and “B,” including the ARGs which were manipulated with known variation among the groups. ERT ranked both *sul*1 and *tet*(W) among the top 10 discriminatory ARGs based on their Gini importance, whereas *erm*B received a low Gini importance score (Fig. 2a). Cluster quality was evaluated using average silhouette score, which improved from 0.08 to 0.65 for the groups (Fig. 2b, c). To provide insight into the profiles of discriminatory ARGs, we compared the top 10 ARGs ranked by ERT as being discriminatory along with the profile of *erm*B that was added at high abundance (Additional file 1: Figure S3). It was observed that the proposed approach was not biased towards the ARGs with high relative abundance. Rather, ERT captured the ARGs with significant variations in their profile (*p* < 0.05). This capability helps overcome the high background occurrence of common housekeeping genes and provides a better resolution into ARG variations.

### Performance

The present ERT with Bayesian optimization (ERT_Bayesian) was compared with existing techniques that are often used for feature selection. The optimal number of features was extracted using different techniques.

**Fig. 3:**
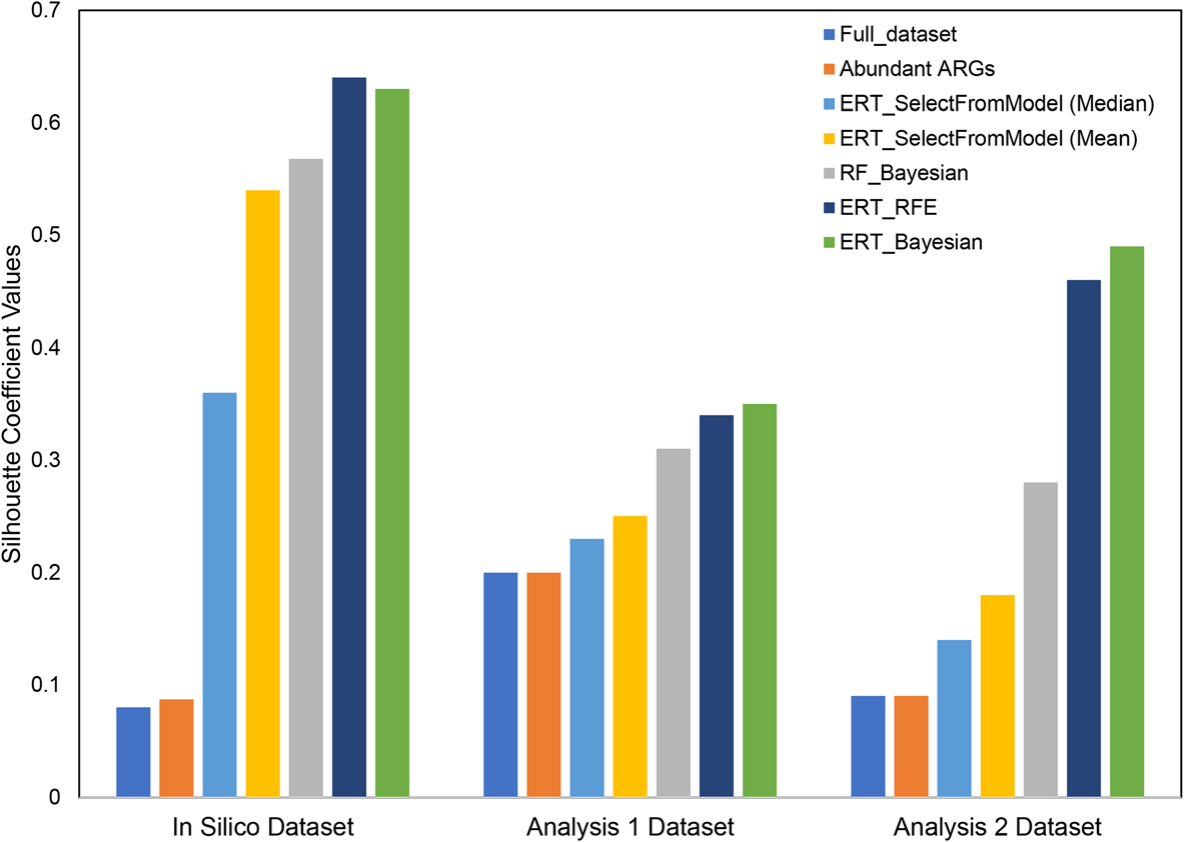
Comparison of silhouette scores estimated using discriminatory features (ARGs) obtained using different classifiers and feature selection methods

Firstly, to illustrate the need for feature selection, we compared the full dataset and most abundant ARGs with the discriminatory ARGs (features) obtained from ERT_Bayesian. Secondly, to elucidate the need for optimization, ERT_Bayesian was compared with the empirical method. The SelectFromModel package from Scikit-learn was used, which is an empirical feature selection method and requires a threshold value to select features. Features are considered important if the corresponding feature importance value is greater than the provided threshold. We used two generic metrics that are the default (mean) and the median-based threshold for this comparison. Thirdly, we compared the RF + Bayesian optimization (RF_Bayesian) with ERT_Bayesian to illustrate the performance of two classifiers. Lastly, Bayesian strategy was compared with a popular wrapper method, i.e., recursive feature elimination (RFE) method [65]. Both the optimization strategies used ERT as the estimator. RFE was implemented using RFECV package from Scikit-learn. All comparisons were done for both simulated and real metagenomic datasets (Fig. 3). Silhouette scores estimated using the discriminatory features were compared.

It was observed that the best performance was obtained by ERT + Bayes and ERT + RFE. Such a result is expected as both the methods were implemented using the same estimator (i.e., ERT) and aim for maximum model performance. However, RFE was very slow in comparison with Bayesian, making a Bayesian optimization faster and overall a better choice. When compared with RF, ERT performed well with all of the datasets. Furthermore, ERT_Bayesian clearly outperformed the empirical techniques in selecting optimal features. Moreover, the number of discriminatory ARGs obtained from other methods was very high, which is suggestive that they are not particularly selective. This defeats the purpose of identifying relevant features from a large dataset such as a metagenomic data, and it does not result in the downscaling of the number of features. With ERT_Bayesian, the optimization step helps downscale the problem and only yields the most discriminatory features. Moreover, the ERT_Bayesian process is fully automated with very little user input. The comparison between abundant and ERT_Bayesian is consistent with the assumption that dominant features are not necessarily the discriminatory features.

### Identification of discriminatory ARGs based upon user-defined labels

#### Analysis 1: Comparison across resistomes (samples from different aquatic environments)

**Fig. 4:**
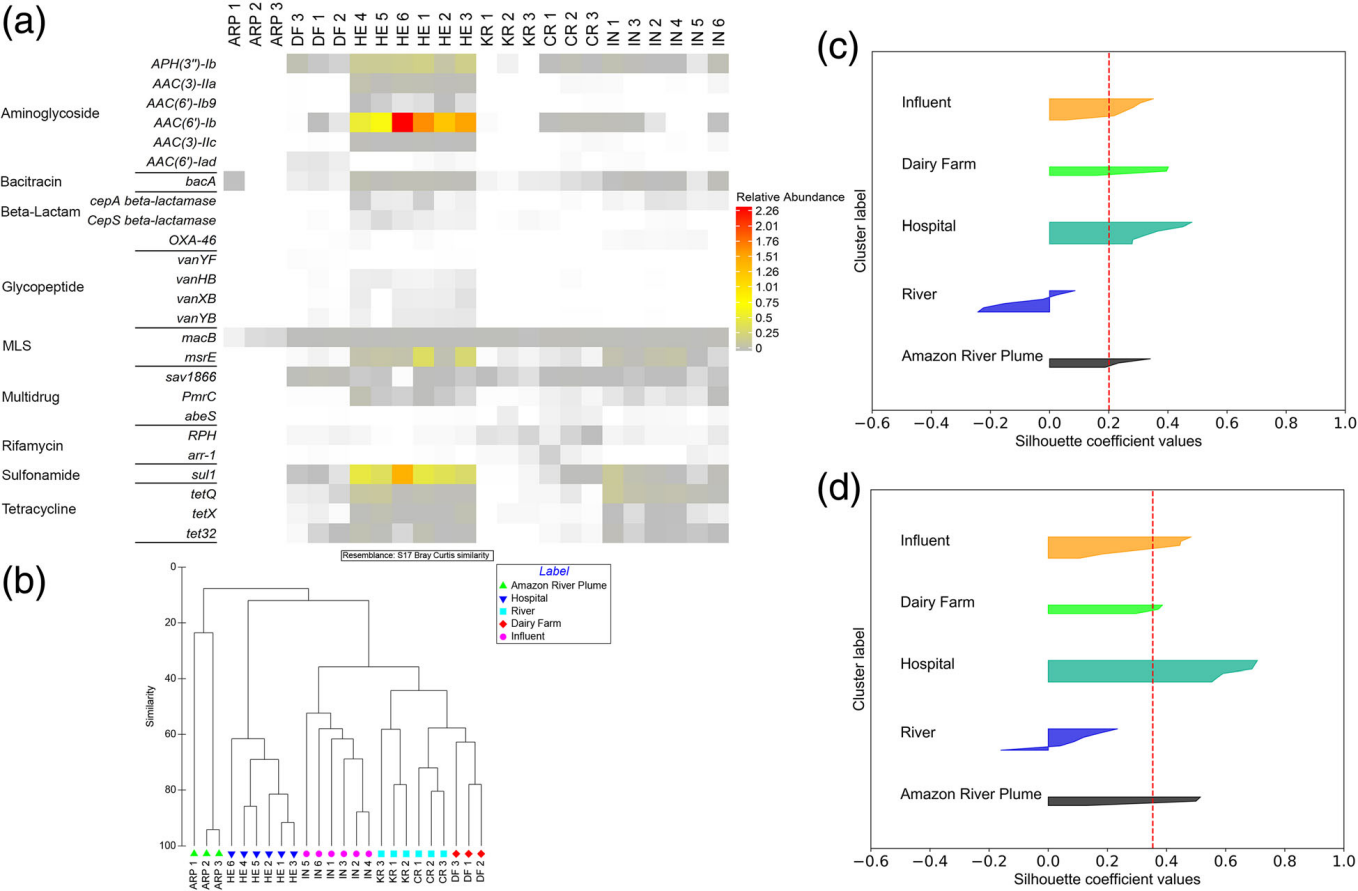
**a** Heatmap and **b** hierarchical clustering of different aquatic environment samples based on the relative abundance of discriminatory ARGs. **c** Silhouette plot for environmental samples using all the annotated ARGs. **d** Silhouette plot for environmental samples using the discriminatory ARGs. (Legend: ARP: Amazon River Plume, DF: Dairy Farm Effluent, HE: Hospital Effluent, KR: Kalamas River, CR: Cam River, IN: Influent)

The first set of metagenomic data analyses served to assess the performance of the established methodology and to validate that the algorithm is effective at distinguishing resistomes representative of a diverse array of environments by identifying discriminatory ARGs. The ERT algorithm was used to generate a list of discriminatory ARGs that effectively classified the resistome characteristic of each environment. The optimal number of discriminatory ARGs, i.e., 36, was selected based on the highest Rand index score (0.87) obtained from the Bayesian optimization. Similarity/dissimilarity analysis using hierarchical clustering (Fig. 4b) and NMDS (Additional file 1: Figure S4) shows that these discriminatory ARGs were able to accurately cluster the samples according to their respective groups. The cluster quality was validated by estimating the sample silhouette coefficient for each label. The analysis showed that the score for each cluster label increased when only the discriminatory ARGs were used to cluster the samples (Fig. 4c, d). The average silhouette score improved from 0.2 to 0.36, thus indicating that the methodology successfully improved the identification of discriminatory ARGs. As observed in Fig. 4b, three major clusters resulted: hospital effluents, Amazon River plume samples, and farm effluent/river samples/WWTP influents. A heatmap of the relative abundances of the top 25 discriminatory ARGs categorized according to the corresponding antibiotic classes in rows and environmental samples in columns provided insight into the occurrence patterns of individual ARGs (Fig. 4a). For example, glycopeptide ARGs had relatively lower abundances in the hospital sewages tested. By contrast, ARGs conferring aminoglycoside (*AAC(6’)-Ib*, *APH(3”)-IB*, *AAC(3)-IIC*, *APH(3)-IIA*, macrolide-lincosamide-streptogramin (MLS; *msrE*), and multidrug resistance *(PmrC)*) were abundant across all the hospital sewages. The identification of a few specific ARGs in the hospital sewages provides evidence that these could be associated with the use of certain specific drugs in the hospitals [66]. Moreover, literature review suggests that the majority of the aminoglycoside resistance genes that were identified here tend to be present within mobile genetic elements (MGEs), such as plasmids, transposons, integrons, and integrative conjugative elements [67], as is the MLS ARG *msrE* [68]. These observations highlight the potential mobility associated with resistomes that are discriminatory or otherwise of interest.

Another observation worth noting was the abundance of *bacA*, often characterized as a housekeeping gene, but also known to confer low-level resistance towards bacitracin in some bacterial genera [69, 70]. This gene was dominant in wastewater influents and hospital sewages, which is not surprising, given that *bacA* is highly characteristic of the human gut [71] and human waste is a major contributor to these samples. The same was observed with respect to tetracycline ARGs (*tetQ*, *tetX*, *tet32*) and the sulfonamide ARG *sul*1 which were also characteristic of both hospital and wastewater influent samples. *tetX* is a flavin-dependent monooxygenase that works by inactivating antibiotics through enzymatic action and is known to confer resistance to all known tetracyclines, especially the broad-spectrum antibiotic tigecycline [72]. Hence, *tetX* is an important candidate for further investigation in terms of its source and fate. On the other hand, *tetQ* and *tet32* confer resistance primarily as ribosomal protection proteins (RPPs), which are often associated with MGEs such as plasmids and transposons [73]. *sul1* is also of prime importance, owing to its association with the resistance genes of class 1 integrons. The rifamycin resistance gene, *arr-*1, a chromosome-encoded ribosyltransferase was only detected in river samples. The aminoglycoside (*AAC(6’)-Iad*) ARG was specifically detected only in farm effluent samples, suggesting that there is a farm-specific characteristic associated with increased loading of this gene type. Notably, these ARGs were not found in the Amazon River plume samples. This is as expected if anthropogenic factors are the main drivers of the observed ARG occurrence patterns, where the Amazonian datasets were selected specifically to represent a low human impact aquatic environment. It was further observed that the aminoglycoside resistance genes (*APH(3”)-Ib* and *AAC(6’)-Ib*) were found in Cam River, but not in Kalamas River samples. These ARGs were also detected in HE 1, HE 2, HE 3, and farm samples. It is important to note that these metagenomic data sets were derived from the same study conducted in Cambridge, UK [19]. It is possible that these different samples might be influenced by each other or by the same site-specific variable resulting in the abundance of specific ARGs in these systems.

#### Analysis 2: Comparison within resistomes (river samples)

In analysis 2, the established ERT methodology was demonstrated for the focused characterization of samples that are similar in nature. Forty-five discriminatory ARGs were identified based on the Gini importance and the Rand index score (= 0.72).

**Fig. 5:**
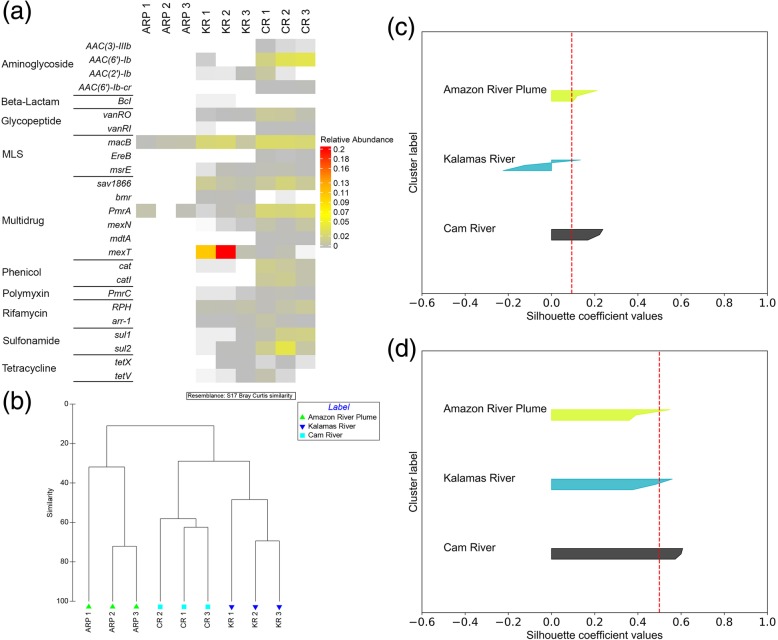
**a** Heatmap and **b** hierarchical clustering of different riverine samples based on the relative abundance of discriminatory ARGs. **c** Silhouette plot for riverine samples using all the annotated ARGs. **d** Silhouette plot for riverine samples using the discriminatory ARGs. (Legend: ARP: Amazon River Plume, KR: Kalamas River, CR: Cam River)

Figure 5b represents the clustering of river resistomes using the discriminatory genes selected using the ERT algorithm. This is supported by the NMDS-based similarity analysis, which indicated increased similarity and improved clustering among samples using discriminatory ARGs (Additional file 1: Figure S5). The mean silhouette coefficient for this analysis increased from 0.09 to 0.45 (Fig. 5c, d), which is consistent with the visual interpretation of the NMDS plot. Out of 45, the top 25 discriminatory ARGs associated with 10 antibiotic classes were visualized using a heatmap (Fig. 5a). Notably, the Amazon River plume was the most distinct when compared with the Kalamas and Cam Rivers. Notably, 23 of the 25 discriminatory ARGs were not detected in Amazon samples, but were present in the Kalamas and Cam samples. These differences in ARG profiles could be due to anthropogenic stressors impacting the river samples from Cambridge and Kalamas. Further, particular ARGs such as aminoglycoside resistance *AAC(3)-IIIb* and *AAC(6')-Ib-cr*, MLS resistance *EreB*, glycopeptide resistance *VanR1*, phenicol resistance *CatI*, and multidrug resistance *mdtA* (membrane fusion protein of the multidrug efflux complex *mdtABC*) were only detected in Cam River, while beta-lactam resistance *BcI* and multidrug resistance *bmr* ARG were only found in Kalamas River. Furthermore, the multidrug resistance gene, *mexT*, which is a regulator of the efflux complex *mexEF-OprN*, was highly abundant in Kalamas River samples, showing a 10 to 20-fold increase when compared with Cam River samples. Previous studies have shown that a number of genes (such as *AAC(6')-Ib*, *AAC(6')-Ib-c*, *msrE*, *sul*1, *sul*2) that were identified as discriminatory have been found to be associated with MGEs. The phenicol ARG, *cat*, which is an umbrella term for many variants of chloramphenicol acetyltransferase has also been observed to be associated with transposons. Interestingly, most of the discriminatory ARGs presumed to be associated with MGEs were not observed in Amazon River plume samples. This observation supports the potential role of MGEs in the dissemination of these ARGs. These are just a few examples of the kinds of patterns that emerged based on examination of the occurrence patterns of the discriminatory ARGs. Overall, the ERT algorithm demonstrated sufficient sensitivity to effectively classify similar environments and identify discriminatory ARGs.

## Discussion

The ERT algorithm was able to effectively identify and classify simulated ARG occurrence variations for both in silico and real metagenomic datasets. The value of in silico data sets for validating methodologies is increasingly being recognized, given inevitable variation that occurs in natural data sets as a result of uncontrollable factors, not excluding DNA extraction efficiency and bias and variable sequencing depth. Further, analysis 1 served to demonstrate the appropriateness of the algorithm for differentiating highly distinct aquatic environments, which clustered according to expectation, and identifying corresponding discriminatory ARGs. It was particularly compelling to find that the hospital metagenomes, which were retrieved from two different studies, displayed high similarity in terms of the specific ARGs that they harbored. This finding supports the notion of a potential “core resistome” associated with hospital wastewater. Here we define a “core resistome” as essentially the opposite of the “discriminatory resistome,” i.e., the ARGs most commonly encountered across a sample set. ARG-MGE associations are well known to occur, and hence, investigations of the core resistome in conjunction with mobilome analysis could lead to better understanding of the potential for ARG dissemination and subsequently inform risk assessment of specific sources [74]. As it is known that wastewater influent and hospital wastewater are associated with human waste, it was further interesting to observe the commonality of high abundance of human-specific ARGs in these samples. While these observations require further validation, these patterns identify potential foci for future research. Analysis applied to very different environments could prove useful in identifying key attributes of corresponding resistomes. This type of analysis could be beneficial in identifying the potential source of the ARGs and in formulating improved surveillance strategies.

Analysis 2, comparing different riverine environments across the globe, further demonstrated that the ERT algorithm has sufficient resolution for distinguishing resistome characteristic of highly similar environments. In addition to relative levels of anthropogenic inputs, site-specific variables such as climatic conditions likely played a role in shaping the ARG profiles [75]. Such analyses could prove to be a stepping stone in identifying the environmental and anthropogenic stressors leading to the proliferation of ARGs. Future studies can adapt the ERT algorithm developed here towards testing various hypotheses of interest. For example, one could frame a study to characterize the effects of each stage of wastewater treatment on ARG occurrence patterns or to characterize baseline geospatial variation in ARG profiles in natural water bodies.

A key advantage of the ERT methodology is the holistic analysis that it provides in a format highly amenable to visual comparison. In particular, it overcomes the bias towards dominant ARGs typical of similarity/dissimilarity analysis and feature projection methods, which can overshadow other insights and lead to the loss of information or an incomplete picture. Moreover, it overcomes the dominant background signal, as demonstrated using in silico datasets. Antibiotic resistance proliferation is a global problem, but it is also greatly influenced by site-specific variables. Both anthropogenic and geospatial variables influence ARG proliferation [76–78]. The combined dynamics of background occurrences, co-selection pressures, temporal variations, and frequency of genetic exchange can further vary the conditions creating an environment that favors specific ARGs [78]. In essence, each variable has individual, synergistic, and antagonistic effects in shaping the resistome. The interconnectivity of various pathways of ARGs and aquatic environments further challenge the ability to delineate sources and mechanisms of ARG dissemination. Under such a multiplexed system, it is crucial to look into the ARGs that are behaving variedly in different environments or different places. Moreover, the method could be extended to the entire set of genes such as MGEs and MRGs for label discrimination and studying co-occurrence patterns. Combined with the validations using qPCR and other methodologies, the effort can lead to an improved understanding of the effect of various stressors. This study demonstrates that the methodology developed here can efficiently target and identify such discriminatory ARGs.

While the developed methodology is quite promising for resistome characterization, it should be noted that there are additional factors that could play a role in data interpretation. Notably, several databases are available for ARG annotation (e.g., SARG [79], DeepARG-DB [80], Comprehensive Antibiotic Resistance Database (CARD)). In this study, CARD was selected because it is well-curated and extensively cited in the literature. For the detection of resistance elements, a protein homolog model reference was used which does not include mutation as a determinant of resistance. Still, it is important to note that, in any metagenomic analysis, annotations inherently will contain some degree of bias based on the database selected, none of which are exhaustive or lacking in erroneous entries. In both analysis 1 and 2, it was observed that in some cases, most of the genes that are a part of an operon were identified as discriminatory ARGs. For example, in analysis 1, *vanHB*, *vanXB*, and *vanYB* all are a part of the *vanB* gene cluster [81] and were identified as discriminatory. In analysis 2, both *mexT* and *OprN* were identified as discriminatory ARGs, where *mexT* is a regulator of *MexEF-OprN* system [82]. However, there were a number of cases where not all the genes of an operon were identified as discriminatory ARGs. For example, *vanRI* and *vanRO* are regulatory proteins associated with glycopeptide resistance gene clusters and were identified as discriminatory, but the same was not observed for other genes belonging to these operons [83, 84]. Similar observation could be made for *mdtA* which is a part of efflux complex *mdtABC* [85]. This result could be attributed to a number of reasons such as annotation parameters, sequencing depths, and sequencing errors. Furthermore, the annotations are based on similarity search, which infers that there could be many ARGs that were missed or incorrectly annotated during the annotations owing to the limited knowledge, computational abilities, and available technologies. For example, *vanRO* sequence is homologous to many other regulatory proteins at an identity of 99% and the same is true for many other *van*-type gene clusters [81]. This brings to light various caveats and potential biases introduced by databases and sequencing platforms that deserve attention in future work. Also, considering the complexity of microbial environments as well as the numerous niches and corresponding anthropogenic pressures, the potential presence of novel or unidentified ARGs is likely. Importantly, the fact that no existing database contains 100% of existing ARGs in nature should be considered when attempting to characterize and differentiate environments.

As new ARGs are continuously being added to the databases, the ARG profiles obtained from different versions of databases could also be different. Since the presented methodology uses the relative abundance metrics of ARGs to identify discriminatory ARGs, it is expected that using different databases could generate different sets of discriminatory ARGs. Hence, to be consistent within a given study, the database version for ARG annotation should be maintained consistent throughout as a precautionary approach, as was the case in the present study. Another important point that needs consideration is potential bias introduced by the metric used to estimate the importance of features. In this study, the Gini importance was applied as a simple, fast, and widely applied means of impurity reduction. However, it should be noted that this method could be biased towards features with multiple possible split points and high minor allele frequency [86].

As is the case with most of the metagenomic data analysis involving public databases, the effectiveness of the method could very well be limited by the underlying differences in sample pretreatment, sample processing, and prior sample contamination. For example, different DNA extraction kits could present their own biases to the samples [87] (Additional file 1: Supplementary Information I). The difference in the sequencing depths and different sequencing platforms might also bias the analyses to a degree as low-coverage samples could lead to misleading inferences. Here we demonstrated the approach both with in silico datasets, where these factors were controlled, and with real-world datasets, where they were not. We judge that efforts towards standardizing approaches and improvements in sequencing power could be key in curbing such biases and drawing effective global-scale comparisons. In particular, improved consistency in the data quality could lead to profound observations using the ERT algorithm approach developed here in identifying discriminatory ARGs. The approach could further be strengthened in its ability by expanding the sample size. It is expected that more concrete patterns will emerge with increases in sample size.

## Conclusions

Here a new methodology was formulated to characterize and compare variances in ARG profiles among metagenomic data sets derived from similar/dissimilar environments. Specifically, identification of discriminatory ARGs among samples representing various environments can be identified based on factors of interest. The proposed methodology presents an effective way to analyze, visualize, and compare environmental resistomes. Ultimately, the ERT approach can offer a new tool for surveillance of environmental ARGs and a means of assessing effectiveness of mitigation strategies.

## Experimental section

### Data sources

In total, 24 shotgun metagenomic sequencing data sets representing a wide range of environments were selected for this study. These included six hospital effluents, nine river source waters, four farm effluents, and six WWTP influents [19, 55, 57, 58]. Among these samples, the hospital effluent, river water, and farm effluent metagenomes were publicly available and downloaded from the EMBL-EBI (https://www.ebi.ac.uk/) and NCBI-SRA (https://www.ncbi.nlm.nih.gov/sra) metagenome databases. WWTP influent metagenomic data was available in-house from a sampling campaign of WWTPs situated in the USA, Switzerland, the Philippines, Sweden, Hong Kong, and India. To maintain uniformity, only datasets generated on the Illumina shotgun sequencing platform were selected. The average number of reads over all the datasets was 13.8 million with a minimum and maximum of 1.3 and 82.8 million reads, respectively. Detailed information about the metagenomes retrieved from the databases is included in Table 1, and the influent data is presented in Table 2.

### Sample collection

WWTP influent samples were collected after the grit removal and screening process. Grab samples were collected from each site and transported to the lab on ice. Biomass from the liquid samples was filter-concentrated onto three separate 0.45-μm filters after homogenizing each sample by shaking. Each membrane filter was then preserved in 50% ethanol at − 20 °C [87] and then shipped to the Molecular Biology Lab at Virginia Tech for DNA extraction and further analyses.

### DNA extraction and shotgun metagenomic sequencing

DNA was extracted from the filter-concentrated samples using a FastDNA Spin Kit (MP Biomedicals, Solon, OH) for soil according to the prescribed protocol. Total DNA was eluted in 100 μL of water and stored at − 20 °C until further analysis. The concentration and quality of extracted DNA were analyzed using NanoPearl spectrophotometry, Qubit fluorometry, and agarose gel electrophoresis. Libraries were prepared using a TruSeq library prep kit, and shotgun metagenomics sequencing was performed on an Illumina HiSeq2500 platform with 2 × 100 paired-end reads by the Virginia Tech Biocomplexity Institute Genomic Sequencing Center, Blacksburg, VA, USA. Two of the samples were duplicated to verify sequencing reproducibility.

### Bioinformatic analysis

FastQ files obtained from shotgun metagenomic sequencing and the public databases were uploaded onto the MetaStorm server to compute the relative abundance of ARGs [52]. The read matching pipeline was used for ARG annotation of the metagenomic data by mapping the raw reads to a reference ARG database using the marker gene analysis approach [88]. This approach uses Diamond [89] with the representative hit approach having *E* value < 10^−10^, identity > 80%, [90], and minimum length of 25 amino acids for the annotation. Sequences were annotated to antibiotic resistance function using the CARD v. 1.0.6 [91]. The database version was consistent throughout the analyses. Further, the samples were compared based on the relative abundance of annotated ARGs, where ARG abundance was normalized based on the total number of 16S rRNA genes present in the sample. This normalization provides an indication of the proportion of bacterial populations carrying the functional genes of interest, though it must be recognized that this is an imperfect measure given that the number of copies of 16S rRNA genes varies per cell. We note that CARD contains various efflux proteins that can be found in both antibiotic resistant and susceptible bacteria and may not be classified as valid markers of resistance phenotypes. In previous studies, however, these were related to efflux of antibiotics and have been classified as ARGs. Accordingly, in this study, efflux proteins were also included in the ARG profiles.

### Statistical analysis

The non-parametric multivariate statistical test PERMANOVA was performed to compare whether the distributions and abundances of ARGs among various environments or defined groups were statistically different. NMDS was conducted on the relative abundance matrix of ARGs obtained from MetaStorm to visualize the level of similarity between the samples in the metadata using the Bray-Curtis similarity method [92]. Firstly, the similarity analysis was done with all the annotated genes obtained from the MetaStorm server and then compared to the NMDS plot generated based on the relative abundance metric of the ARGs selected upon the application of the ERT algorithm. The analysis represents the effectiveness of the ERT algorithm in selecting the ARGs specific to a given environment and in enhancing the characterization of the different environments. All of the statistical analyses were performed using PAleontological STastics software (version 3.18), and NMDS was done using the PRIMER-E Software (v6).

## Additional files

Additional file 1: Supplementary information file. **Figure S1.** Methodology of Data Labeling. The Raw data consists of metagenomic DNA sequence reads derived from different samples. The raw data is labeled according to the user-defined group labels. **Figure S2.**Variable importance determined by the ERT algorithm.This figure illustrates the output of the ERT Algorithm. The Y-axis represents the Gini importance value and the X-axis corresponds to the attributes (in this study, ARGs) sorted in ascending order of their Gini importance. The attribute with the highest Gini importance is most suitable for differentiating samples according to the user-defined group labels, and is ranked first in the list. Similarly, all the attributes are ranked based on their Gini importance score. This plot represents the concept of variable ranking. **Figure S3.** (Left) Profile of identified discriminatory ARGs. (Right) Profile of dominant ARG with no significant variation among the samples. **Figure S4.** (a) NMDS plot for environmental samples using all the annotated ARGs (b) NMDS Plot for environmental samples using the discriminatory ARGs. **Figure S5.** (a) NMDS plot for riverine samples using all the annotated ARGs (b) NMDS Plot for riverine samples using the discriminatory ARGs. (DOCX 1074 kb)

Additional file 2: SI simulated data. (XLSX 32 kb)

## Data Availability

The proposed methodology has been packed into a Python program that can be easily installed. The source code is open and hosted in the Git repository: https://github.com/gaarangoa/ExtrARG. The wastewater influent metagenomic datasets have been deposited in NCBI Short Read Archive (SRA) under the bioproject PRJNA527877.

## Abbreviations

ARG: Antibiotic resistance gene
CARD: Comprehensive Antibiotic Resistance Database
CART: Classification and Regression Trees
ERT: Extremely randomized tree
MGE: Mobile genetic element
MLS: Macrolide-Lincosamide-Streptogramin
NMDS: Non-metric multi-dimensional scaling
PCA: Principal component analysis
PCoA: Principal coordinate analysis
qPCR: Quantitative polymerase chain reaction
RF: Random forest
RFE: Recursive feature elimination
WHO: World Health Organization
WWTP: Wastewater treatment plant
