# Supplementary material for: Identification of discriminatory antibiotic resistance genes among environmental resistomes using extremely randomized tree algorithm
Source: Microbiome. 2019 Aug 29;7:123. doi: 10.1186/s40168-019-0735-1 (PMC6716844; doi:10.1186/s40168-019-0735-1)
Supplement: Supplementary file 1 — Supplementary information file. Figure S1. Methodology of Data Labeling. The Raw data consists of metagenomic DNA sequence reads derived from different samples. The raw data is labeled according to the user-defined group labels. Figure S2.Variable importance determined by the ERT algorithm.This figure illustrates the output of the ERT Algorithm. The Y-axis represents the Gini importance value and the X-axis corresponds to the attributes (in this study, ARGs) sorted in ascending order of their Gini importance. The attribute with the highest Gini importance is most suitable for differentiating samples according to the user-defined group labels, and is ranked first in the list. Similarly, all the attributes are ranked based on their Gini importance score. This plot represents the concept of variable ranking. Figure S3. (Left) Profile of identified discriminatory ARGs. (Right) Profile of dominant ARG with no significant variation among the samples. Figure S4. (a) NMDS plot for environmental samples using all the annotated ARGs (b) NMDS Plot for environmental samples using the discriminatory ARGs. Figure S5. (a) NMDS plot for riverine samples using all the annotated ARGs (b) NMDS Plot for riverine samples using the discriminatory ARGs. (DOCX 1074 kb) [file 40168_2019_735_MOESM1_ESM.docx]

# **Identification of discriminatory antibiotic resistance genes among environmental resistomes using Extremely Randomized Trees algorithm**

## Suraj Gupta^1^, Gustavo Arango-Argoty^2^, Liqing Zhang^2^, Amy Pruden^3^, Peter Vikesland^3^

## ^1^ The Interdisciplinary PhD Program in Genetics, Bioinformatics, and Computational Biology, Virginia Tech, Blacksburg, Virginia 24061, USA

^2^ Department of Computer Science, Virginia Tech, Blacksburg, Virginia 24061, USA

^3^ Via Department of Civil and Environmental Engineering, Virginia Tech, Blacksburg, Virginia 24061, USA

**Supplementary Information**

Supplementary Figure 1: Methodology of Data Labeling. The Raw data consists of metagenomic DNA sequence reads derived from different samples. The raw data is labeled according to the user-defined group labels.

Supplementary Figure 2: Variable importance determined by the ERT algorithm.

This figure illustrates the output of the ERT Algorithm. The Y-axis represents the Gini importance value and the X-axis corresponds to the attributes (in this study, ARGs) sorted in ascending order of their Gini importance. The attribute with the highest Gini importance is most suitable for differentiating samples according to the user-defined group labels, and is ranked first in the list. Similarly, all the attributes are ranked based on their Gini importance score. This plot represents the concept of variable ranking.


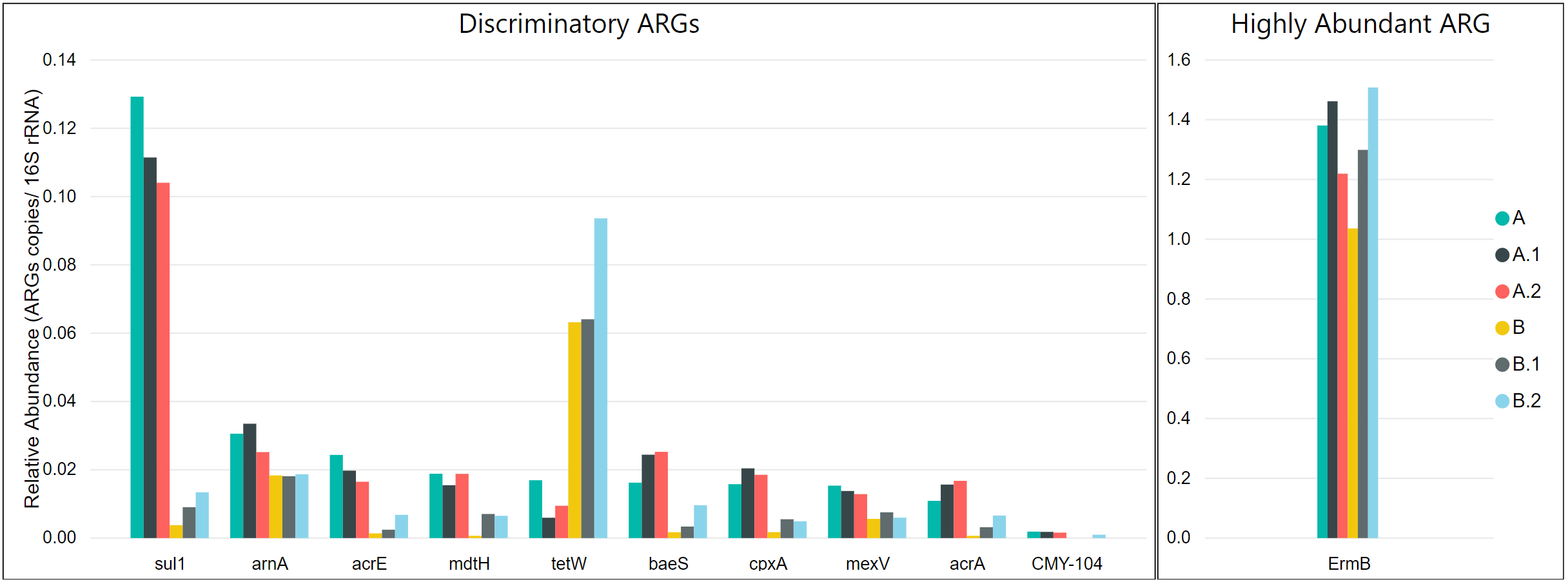


Supplementary Figure 3: (Left) Profile of identified discriminatory ARGs. (Right) Profile of dominant ARG with no significant variation among the samples.


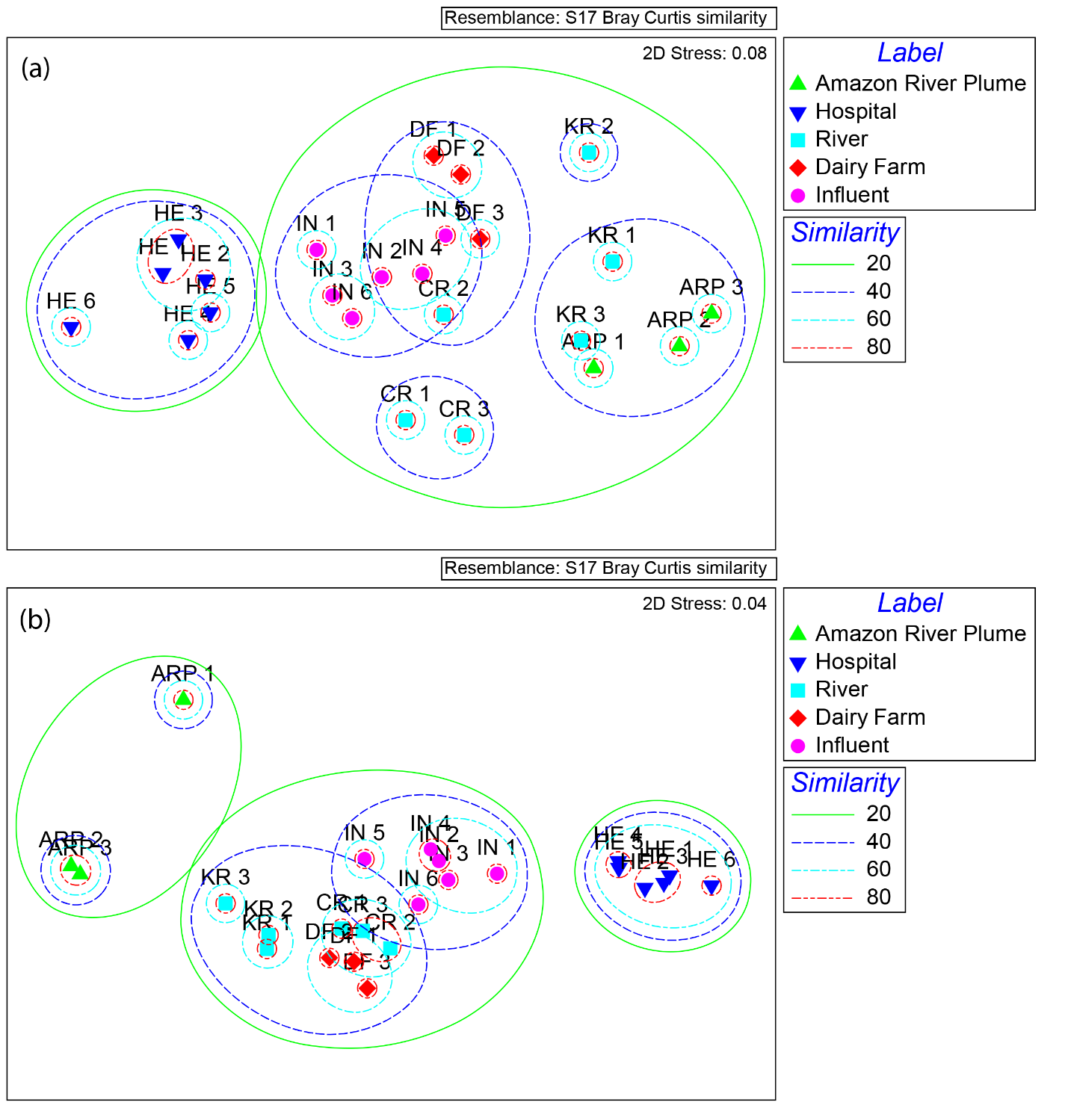


Supplementary Figure 4: (a) NMDS plot for environmental samples using all the annotated ARGs (b) NMDS Plot for environmental samples using the discriminatory ARGs


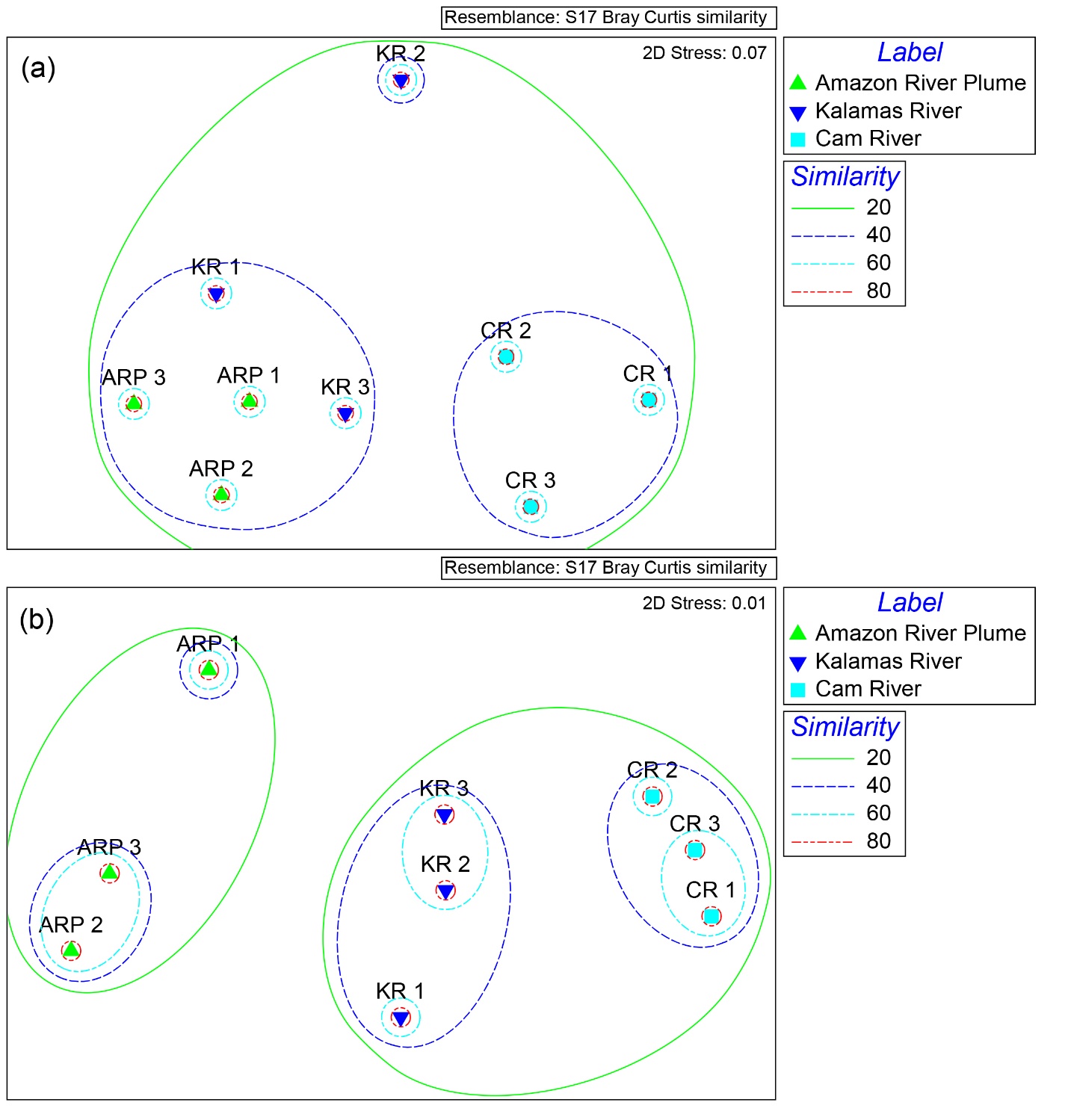


Supplementary Figure 5: (a) NMDS plot for riverine samples using all the annotated ARGs (b) NMDS Plot for riverine samples using the discriminatory ARGs

**Supplementary Information I: Analysis of samples extracted with different DNA Extraction Kit**

In this analysis, the wastewater influent metagenomic samples were extracted using three different DNA extraction kits (i.e., FastDNA Spin Kit (MP) (MP Biomedicals, Solon, OH), PowerSoil DNA Isolation Kit (MO) (MoBio Laboratories, Inc., USA) and ZR fecal DNA Miniprep (ZY) (Zymo Research Corporation, USA)). The samples were extracted from our previous study conducted to examine the effect of sample preservation and different DNA extraction kits on quantification of antibiotic resistance genes in wastewater [1]. We used the methodology described in the main manuscript to identify the differences between these kits in the context of antibiotic resistance. The heatmap (Supplementary Figure 4) comparing the relative abundances of discriminatory ARGs illustrates subtle differences among these kits. For example, *MIR-12* was detected only in MO kit. There were many ARGs such as *AAC(6’)-31, MIR-12, DHA-15, FOX-8, Erm*T*, arr-8, Tri*A that were not detected in the MP kit, but were detected in one or both of MP and ZY kits. Also, the relative abundance of all the discriminatory ARGs was higher among the ZY kit samples when compared with the other two kits. These differences could be due to selective enrichment of specific genes during extraction or difference in the yield and quality of the extraction. Overall, this analysis supports the notion that there could be bias in the comparison of metagenomic datasets which were extracted from different extraction kits.

Supplementary Figure 4: Heatmap of samples extracted using different DNA extraction kits based on the relative abundance of discriminatory ARGs.

**Supplementary Information II:**

The coverage of each sample from the literature used in the present study was examined using the rarefaction curves to minimize biases due to differences in the sequencing depths. These differences could bias the analyses to a degree as low-coverage samples could lead to misleading inferences. Hence, it was imperative to take the necessary step before selecting the samples. It was observed that all the samples achieved sufficient sequencing depths as the rarefaction curves were all nearly plateauing (Supplementary Figure 5).

Supplementary Figure 5: Rarefaction Curves plotted for the metagenomic datasets used in this study

**References**

*1. Li, A.-D., et al., Effects of sample preservation and DNA extraction on enumeration of antibiotic resistance genes in wastewater. 2017.* ***94****(2): p. fix189.*

*2. Meziti, A., Tsementzi, D., Ar. Kormas, K., Karayanni, H. and Konstantinidis, K.T.J.E.m. (2016) Anthropogenic effects on bacterial diversity and function along a river‐to‐estuary gradient in Northwest Greece revealed by metagenomics. 18(12), 4640-4652.*

*3. Ng, C., Tay, M., Tan, B., Le, T.-H., Haller, L., Chen, H., Koh, T.H., Barkham, T.M. and Gin, K.Y.-H. (2017) Characterization of metagenomes in urban aquatic compartments reveals high prevalence of clinically relevant antibiotic resistance genes in wastewaters. Frontiers in microbiology 8.*

*4. Rowe, W.P., Baker-Austin, C., Verner-Jeffreys, D.W., Ryan, J.J., Micallef, C., Maskell, D.J. and Pearce, G.P. (2017) Overexpression of antibiotic resistance genes in hospital effluents over time. Journal of Antimicrobial Chemotherapy 72(6), 1617-1623.*

*5. Satinsky, B.M., Zielinski, B.L., Doherty, M., Smith, C.B., Sharma, S., Paul, J.H., Crump, B.C. and Moran, M.A.J.M. (2014) The Amazon continuum dataset: quantitative metagenomic and metatranscriptomic inventories of the Amazon River plume, June 2010. 2(1), 17.*
